# Supplementary material for: Discovery and application of insertion-deletion (INDEL) polymorphisms for QTL mapping of early life-history traits in Atlantic salmon
Source: BMC Genomics. 2010 Mar 8;11:156. doi: 10.1186/1471-2164-11-156 (PMC2838853; doi:10.1186/1471-2164-11-156)
Supplement: Additional file 2 — Information on developed 76 locus single-run INDEL panel in Atlantic salmon. Information on fluorescence labeling, primer concentrations, PCR pooling and links to alignments, INDEL motifs and GENESCAN (Burge and Karlin 1997) predictions of genes/exons are available in html format. [file 1471-2164-11-156-S2.ZIP › Additionalfile2/snpsummary9520.html]

```
Cluster 3179 Contig 1

prev  Summary    Contig List  next
```

Size of Consensus sequence = 655

Number of sequences = 17

Minimum redundancy = 6

Key

A gi|117299225|gb|EG649037.1|EG649037 SGP259968 Atlantic salmon normalized Muscle cDNA library Salmo salar cDNA clone NM1-1325 5', mRNA sequence  
B gi|117459026|gb|EG791245.1|EG791245 EST\_ssal\_evd\_11440 ssalevd thymus Salmo salar cDNA Salmo salar cDNA clone ssal\_evd\_514\_061\_fwd 3', mRNA sequence  
C gi|15967286|gb|BE518513.2|BE518513 EST00089 Atlantic salmon Lambda Zap Express testis cDNA library Salmo salar cDNA clone TSN-1010 5' similar to Small nuclear ribonucleoprotein, mRNA sequence  
D gi|117459027|gb|EG791246.1|EG791246 EST\_ssal\_evd\_11441 ssalevd thymus Salmo salar cDNA Salmo salar cDNA clone ssal\_evd\_514\_061\_rev 5', mRNA sequence  
E gi|117443092|gb|EG775315.1|EG775315 EST\_ssal\_evd\_50568 ssalevd thymus Salmo salar cDNA Salmo salar cDNA clone ssal\_evd\_568\_031\_fwd 3', mRNA sequence  
F gi|117446284|gb|EG778505.1|EG778505 EST\_ssal\_evd\_5038 ssalevd thymus Salmo salar cDNA Salmo salar cDNA clone ssal\_evd\_505\_150\_fwd 3', mRNA sequence  
G gi|45311832|gb|CK882201.1|CK882201 SGP142564 Atlantic salmon Head kidney cDNA library Salmo salar cDNA clone FN5-0605 5', mRNA sequence  
H gi|24337741|gb|CA038086.1|CA038086 ssallna018040 liver Salmo salar cDNA, mRNA sequence  
I gi|117517680|gb|EG849439.1|EG849439 EST\_ssal\_eve\_6888 ssaleve thyroid Salmo salar cDNA Salmo salar cDNA clone ssal\_eve\_508\_136\_rev 5', mRNA sequence  
J gi|117517681|gb|EG849440.1|EG849440 EST\_ssal\_eve\_6889 ssaleve thyroid Salmo salar cDNA Salmo salar cDNA clone ssal\_eve\_508\_136\_fwd 3', mRNA sequence  
K gi|117470498|gb|EG802717.1|EG802717 EST\_ssal\_evd\_59228 ssalevd thymus Salmo salar cDNA Salmo salar cDNA clone ssal\_evd\_579\_364\_rev 5', mRNA sequence  
L gi|117446285|gb|EG778506.1|EG778506 EST\_ssal\_evd\_5039 ssalevd thymus Salmo salar cDNA Salmo salar cDNA clone ssal\_evd\_505\_150\_rev 5', mRNA sequence  
M gi|117482129|gb|EG814346.1|EG814346 EST\_ssal\_evd\_34231 ssalevd thymus Salmo salar cDNA Salmo salar cDNA clone ssal\_evd\_545\_229\_fwd 3', mRNA sequence  
N gi|24336727|gb|CA037422.1|CA037422 ssallna020032 liver Salmo salar cDNA, mRNA sequence  
O gi|117839803|gb|EG912499.1|EG912499 EST\_ssal\_evf\_14407 ssalevf mixed\_tissue Salmo salar cDNA Salmo salar cDNA clone ssal\_evf\_517\_300\_fwd 3', mRNA sequence  
P gi|117839804|gb|EG912500.1|EG912500 EST\_ssal\_evf\_14408 ssalevf mixed\_tissue Salmo salar cDNA Salmo salar cDNA clone ssal\_evf\_517\_300\_rev 5', mRNA sequence  
Q gi|117482130|gb|EG814347.1|EG814347 EST\_ssal\_evd\_34232 ssalevd thymus Salmo salar cDNA Salmo salar cDNA clone ssal\_evd\_545\_229\_rev 5', mRNA sequence

3 SNPs detected

A B C D E F G H I J K L M N O P Q  cosegregation weighted

292 T - - - - T T T T T T T - - - - -   2/3 66.67
293 A - - - - A A A A A A A - - - - -   2/3 66.67
321 C C T C C C C C C C C C T T T T T   1/3 33.33
